# Chemical Synthesis of Deoxynivalenol-3-β-d-[^13^C_6_]-glucoside and Application in Stable Isotope Dilution Assays

**DOI:** 10.3390/molecules21070838

**Published:** 2016-06-27

**Authors:** Katharina Habler, Oliver Frank, Michael Rychlik

**Affiliations:** 1 Analytical Food Chemistry, Technische Universität München, Alte Akademie 10, D-85354 Freising, Germany; katharina.habler@tum.de; 2 Food Chemistry and Molecular Sensory Science, Technische Universität München, Lise-Meitner-Straße 34, D-85354 Freising, Germany; oliver.frank@tum.de

**Keywords:** modified mycotoxin, deoxynivalenol-3-glucoside, stable isotope dilution assay, LC-MS/MS, beer, labeled standard, synthesis

## Abstract

Modified mycotoxins have been gaining importance in recent years and present a certain challenge in LC-MS/MS analysis. Due to the previous lack of a labeled isotopologue of the modified mycotoxin deoxynivalenol-3-glucoside, in our study we synthesized the first ^13^C-labeled internal standard. Therefore, we used the Königs-Knorr method to synthesize deoxynivalenol-3-β-d-[^13^C_6_]-glucoside originated from unlabeled deoxynivalenol and [^13^C_6_]-labeled glucose. Using the synthesized isotopically-labeled standard deoxynivalenol-3-β-d-[^13^C_6_]-glucoside and the purchased labeled standard [^13^C_15_]-deoxynivalenol, a stable isotope dilution LC-MS/MS method was firstly developed for deoxynivalenol-3-glucoside and deoxynivalenol in beer. The preparation and purification of beer samples was based on a solid phase extraction. The validation data of the newly developed method gave satisfying results. Intra- and interday precision studies revealed relative standard deviations below 0.5% and 7%, respectively. The recoveries ranged for both analytes between 97% and 112%. The stable isotope dilution assay was applied to various beer samples from four different countries. In summary, deoxynivalenol-3-glucoside and deoxynivalenol mostly appeared together in varying molar ratios but were quantified in rather low contents in the investigated beers.

## 1. Introduction

**Figure 1:**
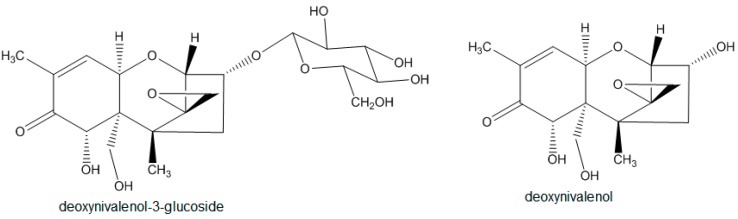
Structures of D3G and DON.

*Fusarium* species are ubiquitously present and can infect a wide range of crops. *Fusarium* head blight, a well-known crop disease caused by different *Fusarium* species, like *F. graminearum*, *F. culmorum*, or *F. avenaceum* [1], can lead to yield loss and reduce grain quality. Fungal infestation of cereals, such as maize, wheat, and barley, is often associated with mycotoxin contamination and, hence, especially affects the safety of human and animal diets. *Fusarium* toxins can be categorized in several major groups: fumonisins, enniatins, zearalenones, and trichothecenes [2]. The latter group can be divided in type A, B, C, and D trichothecenes, among which type A and B toxins are most abundant and relevant in food and feed [2]. Deoxynivalenol (DON) and its modified form deoxynivalenol-3-glucoside (D3G) (Figure 1) rank among the best-known representatives of type B trichothecenes and can present a serious health risk for humans and animals [2].

Maximum limits for unprocessed cereals are legislatively set for DON and zearalenone at 1250 μg/kg and 100 μg/kg, respectively, but have not yet been defined for modified mycotoxins, like D3G [3]. A provisional tolerable daily intake of 1 μg/kg body weight for the sum of DON and its acetylated derivatives was established, but D3G could not be included because of insufficient toxicity and exposure data [4].

More than a decade ago D3G was identified as the most important metabolite of DON and, later, could be found in naturally-contaminated wheat and maize [5,6]. Due to phase-II-metabolism in plants DON can be metabolized and end up as D3G. Moreover, hydrolysis of D3G in human and animal gastrointestinal tracts can again release the aglycone DON [7,8,9], which additionally contributes to the base contamination and plays an emerging issue concerning food safety and health risk assessments. In addition to D3G, several other modified metabolites have already been reported, for example zearalenone-glucoside and -sulfate, nivalenol-glucoside, deoxynivalenol-sulfate, deoxynivalenol-cysteine conjugates, deoxynivalenol-oligo/poly-glucosides, or deoxynivalenol-glutathione adducts [10,11,12,13,14,15,16,17].

Modified mycotoxins are beyond the normal scope of routine analysis, thus leaving them often disregarded unintentionally. However, D3G has already been observed in cereals and barley malt in concentrations up to 19,000 μg/kg [18,19,20,21,22]. In beer samples, the levels of D3G often exceed the levels of DON [23,24] and underlines the urgent need for a substantial risk assessment. Due to the lack of a stable isotope-labeled internal standard, previous studies often quantified D3G by matrix-matched calibration [18,19,20,24]. However, it is generally accepted that isotopologue standards are the tools of choice to compensate for matrix interferences during LC-MS/MS measurements [25].

Due to the lack of a stable isotopologue, the aim of our study was the chemical synthesis of an isotopically-labeled standard of D3G by means of the Königs-Knorr method. We developed a stable isotope dilution assay (SIDA) for D3G and DON by using the newly-synthesized internal standard deoxynivalenol-3-β-d-[^13^C_6_]-glucoside and the commercially available standard [^13^C_15_]-DON, respectively. The thoroughly validated LC-MS/MS method was applied to various beer samples.

## 2. Results

### 2.1. Syntheses

#### 2.1.1. Synthesis of 2,3,4,6-Tetraacetyl-1-bromo-α-d-[^13^C_6_]-glucopyranoside

The acetylation of [^13^C_6_]-glucose was performed with acetic anhydride and pyridine according to Asam and Rychlik [26]. A complete acetylation resulted after 48 h, giving a lightly yellow and clear solution. After a washing step with brine and removal of the solvent, the product 1,2,3,4,6-pentaacetyl-d-[^13^C_6_]-glucopyranoside crystallized during evaporation under cooling in a yield of 87% (0.96 g, 2.4 mmol).

Bromination of the acetylated [^13^C_6_]-glucose (0.5 g, 1.3 mmol) was accomplished with hydrobromic acid in acetic acid following a procedure reported by Ravindranathan Kartha and Jennings, Redemann and Niemann, and Koschella et al. [27,28,29]. The crude product was extracted with dichloromethane followed by successively washing with aqueous sodium thiosulfate, aqueous sodium hydrogen carbonate, and brine. After dissolving the product in diethyl ether and the addition of hexane 2,3,4,6-tetraacetyl-1-bromo-α-d-[^13^C_6_]-glucopyranoside crystallized under cooling as a white compound, yielding 95% (0.5 g, 1.2 mmol).

To obtain enough substance of 2,3,4,6-tetraacetyl-1-bromo-α-d-[^13^C_6_]-glucopyranoside (in total 1.6 g) for the following synthesis of deoxynivalenol-3-β-d-[^13^C_6_]-glucoside, the acetylation and bromination syntheses were repeated four times, respectively.

#### 2.1.2. Synthesis of Deoxynivalenol-3-β-d-[^13^C_6_]-glucoside

Analogously to Savard et al. [30] and Mikula et al. [31,32], for the synthesis of deoxynivalenol-3-β-d-[^13^C_6_]-glucoside the Königs-Knorr method was used. Therefore, the educts DON and 2,3,4,6-tetraacetyl-1-bromo-α-d-[^13^C_6_]-glucopyranoside were mixed with the catalyst silver carbonate in dichloromethane. To facilitate the reaction a surplus of 2,3,4,6-tetraacetyl-1-bromo-α-d-[^13^C_6_]-glucopyranoside and silver carbonate were necessary and hence, were added two further times. Surplus silver ions were precipitated with potassium thiocyanate solution and severed by membrane filtration after reaction.

The controlled hydrolysis of deoxynivalenol-3-2,3,4,6-tetra-*O*-acetyl-β-d-[^13^C_6_]-glucoside to deoxynivalenol-3-β-d-[^13^C_6_]-glucoside was performed in 5 mL acetonitrile/water (1/1, *v*/*v*) with potassium hydroxide. The stoichiometrically-required amount of potassium hydroxide was added dropwise under continuous pH control via pH meter. Due to instability of type B trichothecenes in alkaline range [33], the addition of potassium hydroxide took place drop by drop all day long to guarantee a pH value below eleven. On the next day the hydrolysis reaction was stopped by neutralization with hydrochloride acid before preparative purification.

The synthesis procedure was performed 72 times to obtain a sufficient substance amount as upscaling studies of the reaction resulted in a lower yield.

The recorded ^1^H-NMR and ^13^C-NMR spectra of deoxynivalenol-3-β-d-[^13^C_6_]-glucoside show the following signals:

^1^H-NMR (500 MHz, deuterium oxide) δ 6.65 (dd, *J* = 5.8, 1.3 Hz, 1H, H-10), 4.95 (d, *J* = 5.8 Hz, 1H, H-11), 4.54 (dd, *J* = 10.9, 4.4 Hz, 1H, H-3), 5.00–4.00 (s, 1H, H-7, d, 1H, H-2), 5.00–3.00 (m, 7H, H-1′, H-2′, H-3′, H-4′, H-5′, H-6′a, H-6′b), 4.00–3.00 (d, 1H, H-13a, d, 1H, H-13b, d, 1H, H-15a, d, 1H, H-15b), 2.50–1.50 (dd, 1H, H-4a, dd, 1H, H-4b), 1.91 (s, 3H, -CH_3_-16), 1.12 (s, 3H, -CH_3_-14).

^13^C-NMR (125 MHz, deuterium oxide) δ 202.2 (1C, C-8), 138.1 (1C, C-9), 137.0 (1C, C-10), 97.4 (1C, C-1′, m), 82.0–60.4 (6C, C-3, C-2, C-4, C-7, C-11, C-15), 75.1–76.4 (2C, C-3′, C-5′, m) 73.0 (1C, C-2′, m), 69.5(1C, C-4′, m), 60.7 (1C, C-6′, m), 46.0 (1C, C-13), 14.6 (1C, C-16), 13.9 (1C, C-14).

NMR experiments of deoxynivalenol-3-β-d-[^13^C_6_]-glucoside were performed only with a focus on assigning the glycosidic bond between C-3 of DON and C-1′ of glucose, which could be verified. For quantitation via qNMR [34] the signal of deoxynivalenol-3-β-d-[^13^C_6_]-glucoside at 6.65 ppm (H-10) was chosen. The molar concentration was 0.6106 mmol/L. The yield was 1% (170 μg, 0.37 μmol).

**Figure 2:**
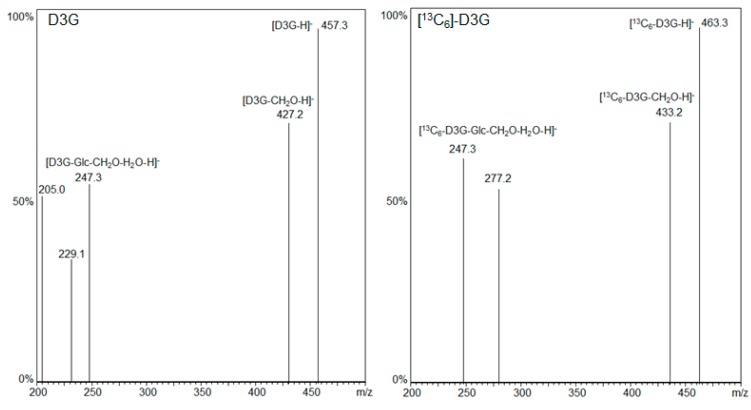
LC-MS/MS fragmentation spectra of D3G and [^13^C_6_]-D3G.

LC-MS/MS fragmentation spectra of D3G and [^13^C_6_]-D3G are displayed in Figure 2. LC-MS/MS measurements were carried out in the negative ESI mode and the respective [M − H]^−^ ions as precursor ions were used. D3G (*m/z* 457.3) and [^13^C_6_]-D3G (*m/z* 463.3) likewise showed the loss of the -CH_2_OH group at position C-6 of the DON molecule resulting in the most intensive fragment for D3G (*m/z* 427.2 = 457.3 − 30) and [^13^C_6_]-D3G (*m/z* 433.2 = 463.3 − 30) as Berthiller et al. [35] already reported for D3G. A further loss of water and glucose or [^13^C_6_]-glucose followed, respectively (D3G *m/z* 247.3 = 457.3 − 30 − 18 − 162, [^13^C_6_]-D3G *m/z* 247.3 = 463.3 − 30 − 18 − 168). For D3G, the transitions (*m/z* 457.3 → 427.2) and (*m/z* 457.3 → 247.3), and for [^13^C_6_]-D3G the transitions (*m/z* 463.3 → 433.2) and (*m/z* 463.3 → 247.3), served as quantifier and qualifier, respectively.

### 2.2. Method Development

#### 2.2.1. LC-MS/MS

DON and [^13^C_15_]-DON were measured in the positive ESI mode. Therefore, the protonated molecules were used as precursor ions, respectively. D3G was analyzed in the negative ESI mode and the abundant [M − H]^−^ ion was used as precursor ion. As already described, the labeled standard [^13^C_6_]-D3G gave a fragmentation pattern similar to the respective unlabeled compound. The LC-MS/MS parameter of the SIDA of D3G and DON are shown in Table 1. A chromatographic separation of D3G and DON could be assured by using a Hydrosphere RP-C18 column (YMC Europe GmbH, Dinslaken, Germany) combined with a specific gradient.

#### 2.2.2. Calibration and Quantitation

Response functions were obtained using linear regression. The response factors were 1.1 for D3G and DON, respectively. The coefficients of determination (*R^2^*) exceeded 0.9994 and as confirmed by Mandel test, the calibration curves showed linearity for D3G and DON within the molar ratios 0.1–10, respectively.

#### 2.2.3. Sample Purification

The purification of beer samples was based on a solid phase extraction. Due to the already developed multi-mycotoxin method of cereals [18], the application of Bond Elut Mycotoxin cartridges (Agilent Technologies, Santa Clara, CA, USA) were again selected.

**Figure 3:**
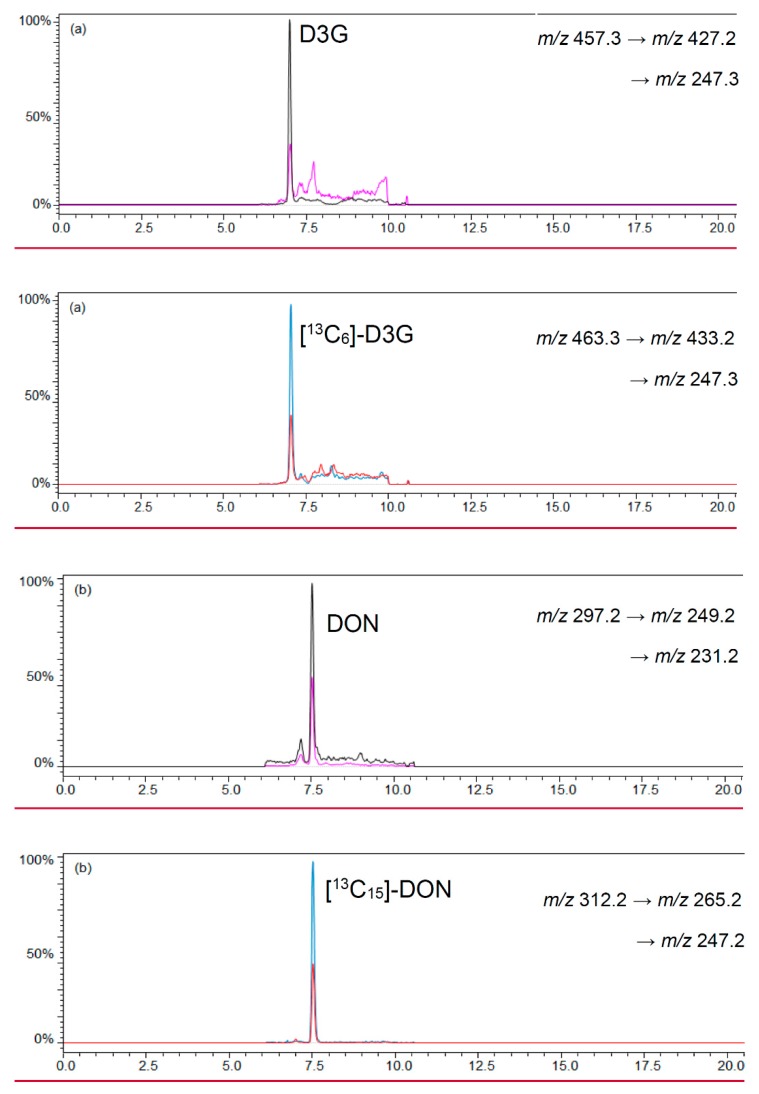
LC-MS/MS chromatograms of (**a**) D3G in negative ESI mode; and (**b**) DON in positive ESI mode in a naturally-contaminated beer sample with 10.6 μg/L D3G and 20.3 μg/L DON.

A ratio of 0.8 mL beer and 4 mL acetonitrile was chosen to precipitate polar matrix compounds in beer and to follow the recommendations of the manufacturer for loading the cartridge [36,37]. Polar matrix compounds in beer were precipitated after mixing the chosen beer and an acetonitrile volume of 0.8 mL and 4 mL, respectively. An overload of the cartridges was not observed with a total sample volume of 4.8 mL. After evaporation of the eluates until dryness and reconstitution of the sample with 200 μL acetonitrile/water (1/1, *v*/*v*) a lightly yellow colored clear sample resulted. The LC-MS/MS chromatograms of beer samples revealed low noise signals that did not interfere or overlap with the signals of the analytes and labeled standards. Figure 3 shows a LC-MS/MS chromatogram of D3G and DON as well as their labeled standards [^13^C_6_]-D3G and [^13^C_15_]-DON in a naturally-contaminated beer sample.

### 2.3. Method Validation

#### 2.3.1. Limits of Detection and Quantitation

The LODs and LOQs for the SIDAs were calculated according to Vogelgesang and Hädrich [38]. Beer free of the monitored mycotoxins was used as blank matrix. The LOD and LOQ were 2.99 μg/L and 8.84 μg/L for D3G and 1.49 μg/L and 4.44 μg/L for DON, respectively (Table 2).

#### 2.3.2. Recovery

The recoveries of the SIDAs were determined at three different spiking levels for each mycotoxin. The recoveries ranged between 112% and 105% for D3G and between 97% and 108% for DON with relative standard deviations (RSDs) below 3% (Table 2).

#### 2.3.3. Precision

The intra-day (*n* = 3) and inter-day (*n* = 3) coefficients of variation are shown in Table 2. The intra-day precision ranged between 0.3% and 0.5% and the inter-day precision ranged between 5% and 7%.

### 2.4. Analysis of D3G and DON in Beer Samples

Different beer samples bought in 2015 from a local market in Munich were analyzed with the SIDA of D3G and DON presented. The nine different beers originated from four different countries, namely Germany, Taiwan, China, and the USA. Apart from one organic wheat beer with 5.1 vol % alcohol, the other conventional beers had an alcohol content varying between 4.7 vol % and 8.3 vol %. The origins, alcohol contents, and the contents of D3G and DON of the analyzed beer samples are summarized in Table 3. On average, the investigated beers were contaminated with 22.6 μg/L D3G and 13.3 μg/L DON. D3G was found with concentrations between 8.31 μg/L and 63.3 μg/L in six beer samples. Seven of the nine analyzed beer samples were contaminated with DON between 5.11 μg/L and 28.3 μg/L. The molar ratios between DON and D3G ranged between 0.34 and 1.54.

## 3. Discussion

### 3.1. Syntheses

In addition to the Königs-Knorr method [39], the Schmidt method [40] also offers a possible synthetic route for glycosylations. In our study, we decided to synthesize deoxynivalenol-3-β-d-[^13^C_6_]-glucoside according to the Königs-Knorr synthesis based on the educts DON and 2,3,4,6-tetraacetyl-1-bromo-α-d-[^13^C_6_]-glucopyranoside with the catalyst silver carbonate in dichloromethane.

The synthesis of 2,3,4,6-tetraacetyl-1-bromo-α-d-[^13^C_6_]-glucopyranoside provided an easy reaction in very good yield above 85%. In contrast, the glycosylation reaction and especially the hydrolysis of the acetyl groups presented a bigger challenge. Additionally, dichloromethane, acetonitrile as an organic solvent, as well as different catalysts like silver carbonate, silver oxide, and silver triflate were tested. All reaction experiments revealed that no product was formed with silver triflate independent of the organic solvent. The synthesis was also performed with molecular sieves (3 and 4 Å) and under argon atmosphere again yielding no product. Better yields were achieved by using silver carbonate and dichloromethane instead of silver oxide and acetonitrile. However, a crucial aspect for higher yields turned out to be the increase of the equivalents of 2,3,4,6-tetraacetyl-1-bromo-α-d-[^13^C_6_]-glucopyranoside and silver carbonate from two to twenty, compared to DON.

Due to the instability of DON under alkaline conditions [33], several hydrolysis variations were considered [30,31,32,41]: potassium carbonate in methanol; potassium cyanide in methanol; potassium hydroxide in water, methanol, tetrahydrofuran/water, and acetonitrile/water. Finally, the deprotection of the acetylated product deoxynivalenol-3-2,3,4,6-tetra-*O*-acetyl-β-d-[^13^C_6_]-glucoside to deoxynivalenol-3-β-d-[^13^C_6_]-glucoside under continuous pH-value control via pH meter with potassium hydroxide in acetonitrile/water emerged as the most practicable solution.

An option to prevent the formation of deoxynivalenol-15-β-d-[^13^C_6_]-glucoside, 15-acetyldeoxynivalenol with its hydroxyl group at C-15 being protected could have been used as the educt. However, as the hydrolysis of the acetyl groups has already presented a problem, it appeared more reasonable to purify the synthesis and separate [^13^C_6_]-D3G from [^13^C_6_]-D15G, DON, and other undesirable products chromatographically by preparative HPLC.

**Figure 4:**
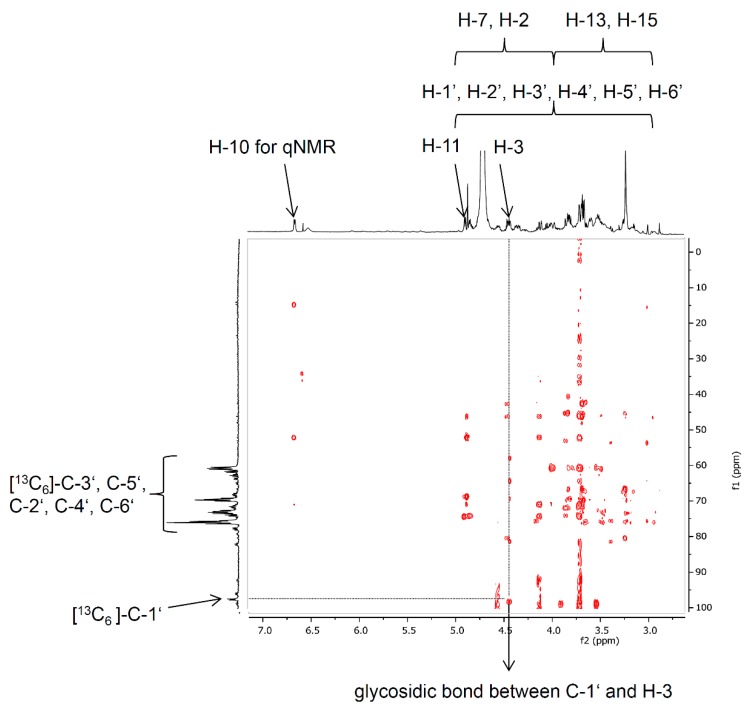
Section of the HMBC NMR spectrum of [^13^C_6_]-D3G showing indicating the glycosidic bond of ^13^C-1′ from [^13^C_6_]-glucose to H-3 of DON.

The structural elucidation of the product [^13^C_6_]-D3G was performed by NMR and especially by the fragmentation pattern after LC-MS/MS measurements. For two reasons, a complete assignment of all NMR signals was not feasible: (a) ^13^C-labeled compounds show intense signals and complex coupling behavior of ^1^H and ^13^C during NMR analysis in combination with (b) the small amount of [^13^C_6_]-D3G (170 μg). However, the ^1^H-NMR signals of H-10, H-11, and H-3, as well as the protons of the methyl groups at C-16 and C-14 of [^13^C_6_]-D3G could be clearly assigned. They showed similar shifts as Blackwell et al. [42] have already reported for DON. The ^13^C-NMR experiment revealed the expected shifts of the keto-group C-8 at 202.2 ppm, the olefinic carbon C-10 resonating at 137.0 ppm as well as the shifts of the carbon atoms C-9, C-13, C-15, and C-14 of the DON structure. The glucose moiety could be easily assigned by the characteristic coupling pattern of the signals with typical ^1^*J*_cc_ coupling constants between 42 and 46 Hz. By means of 2D-experiments (HMBC optimized for ^2,3^*J*_H,C_ couplings) the key correlation signal between C-1′ (97.4 ppm) and H-3 (4.54 ppm) could be observed and, hence, the glycosidic bond was verified via NMR (Figure 4). For application as an isotopically-labeled standard, the unequivocal quantitation of [^13^C_6_]-D3G via qNMR of the resolved signal of H-10 provided the most accurate quantitation of such small amounts.

### 3.2. Method Development

As already described in Habler and Rychlik [18], D3G had the same fragmentation pattern as DON due to in-source fragmentation and loss of glucose from D3G in the positive ESI mode. Therefore, a chromatographic baseline separation of these analytes was essential to allow quantification of DON instead of the sum of D3G and DON in the positive ESI mode. This was achieved using a Hydrosphere RP-C18 column (YMC Europe GmbH, Dinslaken, Germany) as the stationary phase combined with a moderately shallow gradient of 11.25% increase of acetonitrile per minute. To enable optimal MS conditions and sensitivity for both analytes, two chromatographic runs were performed for each sample: The first one was run for D3G in the negative ESI mode without additives in the mobile phase and the second one for DON in the positive ESI mode with formic acid in the mobile phase.

Sample purification using solid phase extraction was designed to reduce consumption of labeled internal standard and to maximize analyte sensitivity. Moreover, a clean-up step is important to extend the HPLC column lifetime and to maintain performance of the ESI-MS source. After addition of acetonitrile to beer samples interfering polar matrix compounds can be precipitated as is already often applied [23,43,44,45]. However, a precipitation of polar mycotoxins, along with the matrix and a resulting poor recovery rate is avoided by using a small sample volume of 0.8 mL beer combined with using the isotopologues [^13^C_6_]-D3G and [^13^C_15_]-DON as internal standards. A sufficient sensitivity would not have been achieved with our instrumentation when using a dilute-and-shoot approach as described by Al-Taher et al., Hu et al., and Malachova et al. [46,47,48].

To be able to expand the method to a multi-mycotoxin stable isotope dilution method including non-polar *Fusarium* toxins, like type A trichothecenes, zearalenone, and enniatins, a SPE-cartridge without activated carbon was used. Otherwise, non-polar mycotoxins would adsorb onto multifunctional SPE-cartridges usually containing activated carbon. Likewise to be able to include other mycotoxins in the developed method the samples were reconstituted with acetonitrile/water in a volume ratio of 1 to 1.

### 3.3. Method Validation

The LODs and LOQs of the presented method for D3G (2.99 μg/L and 8.84 μg/L) and DON (1.44 μg/L and 4.44 μg/L) were similar to those reported by Kostelanska et al. [43] in beer matrix and Nathanail et al. [20] in cereals. Thus, the sensitivity of our method is sufficiently low to quantitate D3G and DON in natural contaminated beers despite of the small sample volume of 0.8 mL. The intra-day and inter-day precisions, with maximum relative standard deviations of 0.5% and 7%, respectively, were similar or even better to previous studies [43,48]. As expected for SIDAs, the recoveries of the newly developed SIDA for D3G and DON ranged between 97% and 112% and, hence, were close to 100%. Due to the previous lack of a stable isotopologue, D3G was often quantified via matrix-matched calibration resulting in rather dissatisfactory recoveries between 39% and 117% [18,43,48]. Thus the validation data of the stable isotope dilution LC-MS/MS method for D3G and DON proved its suitability for further applications.

### 3.4. Analysis of D3G and DON in Beer Samples

The newly-developed SIDA for D3G and DON was firstly applied to beer samples from different countries (Germany, Taiwan, China, USA) with varying alcohol contents between 4.7% and 8.3%. On average, 78% and 89% of the investigated beer samples were found to be contaminated, in detail with 22.6 μg/L D3G and 13.3 μg/L DON, respectively. Except for one IPA from the US, the contents of D3G and DON were rather low and at a maximum of 20 μg/L, respectively. This IPA with the highest alcohol content of 8.3% revealed 63.3 μg/L D3G and 28.3 μg/L DON. The maximum permitted content of D3G and DON in beer has to be derived from the legislatively-set limit for DON in unprocessed cereals of 1250 μg/kg, that is applicable to brewing malt [3]. The maximum permitted content of DON in beer would be 225 μg/L (1250 μg/kg × 18%) calculated by the charge of normal lager. The contents of the investigated beer samples were at least five times below that limit for D3G (calculated as DON) and eight times for DON. Due to the limited number of analyzed beer samples, our study does not allow a risk assessment concerning organic and conventional beers or a relation between alcohol content and contamination level like Harcz et al. [49] and Kostelanska et al. [43] did. Other studies that investigated D3G and DON in beer revealed similar incidences and contents for D3G between 1.2 μg/L and 89.3 μg/L and, for DON, between 1.0 μg/L and 81.3 μg/L [24,43,50]. In our study, D3G and DON could often be detected together. The molar ratios of D3G/DON were between 0.34 and 1.54, but mostly above 1.0, which implies that the levels of D3G exceed the levels of DON. In contrast, Varga et al. [24] and Kostelanska et al. [43] reported molar ratios of D3G/DON between 0.11 and 1.25 (averaged 0.56).

This newly-developed LC-MS/MS method for D3G and DON based on a SIDA provides an easy approach to analyze all kinds of beer. With the synthesized labeled standard of D3G a manifold of beer samples, as well as barley malt and other cereals, can be investigated via SIDA in prospective studies. This is necessary to gain exact and sufficient exposure and toxicity data to be able to derive a justified maximum limit for D3G in food and feed.

## 4. Materials and Methods

### 4.1. Chemicals and Reagents

Acetonitrile, water (both analytical grade), diethyl ether, hydrochloric acid, potassium thiocyanate, sodium chloride, sodium hydrogen carbonate, sodium sulfate, and sodium thiosulfate were purchased from VWR (Ismaning, Germany). DON, formic acid (>95%), silver carbonate, hexane, pyridine, acetic anhydride, hydrogen bromide in acetic acid (33 wt %), deuterium oxide, and deuterated chloroform were bought from Sigma Aldrich (Steinheim, Germany). Dichloromethane was purchased from Merck (Darmstadt, Germany) and [^13^C_6_]-glucose was aquired from Euriso-Top (Saarbrücken, Germany). The unlabeled reference compounds D3G and DON, as well as the labeled standard [^13^C_15_]-DON, were bought from Coring System Diagnostix (Gernsheim, Germany).

### 4.2. Syntheses

#### 4.2.1. Synthesis of 2,3,4,6-Tetraacetyl-1-bromo-α-d-[^13^C_6_]-glucopyranoside

2.65 mL acetic anhydride (2.85 g, 28 mmol, 10 eq.) and 2.25 mL pyridine (2.20 g, 28 mmol, 10 eq.) were added to [^13^C_6_]-glucose (0.52 g, 2.8 mmol, 1 eq.). The mixture was stirred at room temperature for 48 h until the solution was clear. The solution was poured in 10 mL ice cold water and the product was extracted three times with 10 mL dichloromethane, respectively. The combined organic phases were washed with 10 mL brine and dried over sodium sulfate. Dichloromethane was evaporated and 1,2,3,4,6-pentaacetyl-d-[^13^C_6_]-glucopyranoside was stored at −20 °C in the dark.

While cooling, 2.5 mL hydrogen bromide (33 wt % in acetic acid, 5 mL/g glucose) were added to 1,2,3,4,6-pentaacetyl-d-[^13^C_6_]-glucopyranoside (0.5 g, 1.3 mmol). Until the solution was clear, stirring was performed under cooling. Afterwards, the solution was stirred at room temperature for further 6 h. The solution was poured on 10 mL ice cold water and extracted three times with 10 mL dichloromethane, respectively. The combined organic phases were washed once with 10 mL sodium thiosulfate solution (0.5%), three times with 10 mL saturated sodium hydrogen carbonate solution, respectively, and once with 10 mL brine. The organic phase was dried over sodium sulfate and evaporated to dryness. The residue was solved in diethyl ether and overlaid with hexane. After crystallizing under cooling, the solvents were removed under a gentle nitrogen stream and the product was stored at −20 °C in the dark. After conducting these syntheses four times, NMR measurements in deuterated chloroform were performed.

#### 4.2.2. Synthesis of Deoxynivalenol-3-β-d-[^13^C_6_]-glucoside

DON (0.25 mg, 0.85 μmol) was dissolved in 0.3 mL dichloromethane. To the solution 2,3,4,6-tetraacetyl-1-bromo-α-d-[^13^C_6_]-glucopyranoside (0.007 g, 17 μmol) and silver carbonate (0.004 g, 15 μmol) were added and stirred at room temperature in the dark. Another portions of 2,3,4,6-tetraacetyl-1-bromo-α-d-[^13^C_6_]-glucopyranoside (0.007 g, 17 μmol) and silver carbonate (0.004 g, 15 μmol) were added two times after 24 h, respectively. The reaction was stirred for a further three days at room temperature in the dark. The solution was membrane filtrated (0.45 μm) and the solvent was removed under a nitrogen stream at 50 °C. Then the synthesis was reconstituted with 1 mL potassium thiocyanate solution (0.5%) to precipitate surplus silver ions. After membrane filtration (0.45 μm) 2.5 mL ACN and 1.5 mL H_2_O were added to the solution of the protected target compound.

For hydrolysis of DON-2,3,4,6-tetra-acetyl-[^13^C_6_]-glucoside to give DON-3-[^13^C_6_]-glucoside potassium hydroxide (0.012 g, 215 μmol) was dissolved in 0.5 mL H_2_O and was added dropwise under pH control by pH meter to the DON-2,3,4,6-tetra-acetyl-[^13^C_6_]-glucoside solution. The pH was not allowed to exceed 11. The reaction mixture was stored overnight at 5 °C and was neutralized thereafter with 1 M and 0.1 M hydrochloride acid (around four drops each). The synthesis procedure was performed 72 times.

The product DON-3-[^13^C_6_]-glucoside was separated from DON-15-[^13^C_6_]-glucoside, DON, and other side products and purified via preparative HPLC. After combining and removing the solvent of these fractions containing DON-3-[^13^C_6_]-glucoside NMR and qNMR experiments in deuterium oxide, as well as LC-MS/MS measurements were performed.

### 4.3. Preparative HPLC

Preparation and purification of DON-3-[^13^C_6_]-glucoside was carried out on a Merck Hitachi system (Kyoto, Japan) consisting of two pumps (L-7100, Merck Hitachi, Tokyo, Japan), a degasser (Degassex model DG 4400, Phenomenex, Aschaffenburg, Germany), an autosampler (L-7200, Merck Hitachi, Tokyo, Japan), and a UV detector (L-7400, Merck Hitachi, Tokyo, Japan). As the stationary phase a Pro Pack RP-C18 column (150 mm × 10 mm, S-5 μm, 12 nm, YMC Europe GmbH, Dinslaken, Germany) combined with a widepore C18-guard column (4 mm × 3 mm, Security Guard, Phenomenex, Aschaffenburg, Germany) was used. The binary gradient system consisted of (A) water and (B) acetonitrile at a flow rate of 1 mL/min. The shallow gradient to separate DON-3-[^13^C_6_]-glucoside from DON-15-[^13^C_6_]-glucoside, DON, and other side products was raised linearly from 10% B to 100% B during the next 20 min, and then maintained at 100% B for 5 min. Next, the mobile phase returned to 10% B within 2 min and the system was equilibrated for 10 min before the next run. The detection was performed at 218 nm and the injection volume was 100 μL.

### 4.4. NMR and qNMR

The NMR and qNMR measurements were performed on a Bruker AV III system (400 MHz, Bruker, Rheinstetten, Germany) and on a Bruker AV III system (500 MHz, Bruker, Rheinstetten, Germany), respectively. ^1^H- and ^13^C-NMR spectra were recorded at 400 or 500 MHz and at 100.61 or 125.76 MHz, respectively. Data acquisition was performed with TopSpin software 3.2 (Bruker, Rheinstetten, Germany) and MestReNova software 10.0 (Mestrelab Research, La Coruña, Spain). The samples were dissolved in 600 μL or 300 μL deuterium oxide or deuterated chloroform. The qNMR measurement of DON-3-[^13^C_6_]-glucoside was performed as published by Korn et al. [34]. In brief, the compound was dissolved in 600 μL deuterium oxide and analyzed in 5 × 178 mm NMR tubes (Bruker, Rheinstetten, Germany). As an external standard a caffeine sample with known concentration was used.

### 4.5. Preparation of Standard Solution

Stock solutions of labeled and unlabeled toxins were prepared in a concentration of 10 μg/mL in acetonitrile and further diluted to a final concentration of 1 μg/mL. All solutions were stored at 4 °C in the dark.

### 4.6. LC-MS/MS

LC-MS/MS was carried out on a Shimadzu LC-30A Prominence system (Shimadzu, Kyoto, Japan) with a Hydrosphere RP-C18 column (150 mm × 3.0 mm, S-3 μm, 12 nm, YMC Europe GmbH, Dinslaken, Germany) and a C18-guard column (Phenomenex, Aschaffenburg, Germany) as the stationary phase that was kept at 40 °C. The binary gradient system consisted of (A) water and (B) acetonitrile for D3G and of (A) 0.1% formic acid and (B) acetonitrile with 0.1% formic acid for DON at a flow rate of 0.2 mL/min. The gradient for D3G was started and held at 10% B for 1 min, raised linearly from 10% B to 100% B during the next 6 min, and then maintained at 100% B for 4 min. Next, the mobile phase returned to 10% B within 2 min and the system was equilibrated for 10 min before the next run. The gradient for DON was started and held at 10% B for 1 min, raised linearly from 10% B to 100% B during the next 8 min, and then maintained at 100% B for 4 min. Next, the mobile phase returned to 10% B within 2 min and the system was equilibrated for 10 min before the next run. The injection volume was 5 μL.

The LC was interfaced with a triple quadrupole ion trap mass spectrometer (LCMS-8050, Shimadzu, Kyoto, Japan). It operated in the negative ESI mode for the analyte D3G and the internal standard [^13^C_6_]-D3G and in the positive ESI mode for the analyte DON and the internal standard [^13^C_15_]-DON. The ion source parameters were set as follows: interface temperature 300 °C, heat block temperature 400 °C, dilution line temperature 250 °C, heating gas flow 10 L/min, drying gas flow 10 L/min, nebulizing gas flow 3 L/min, collision-induced dissociation gas 17 kPa, interface voltage 4 kV. MS parameters were optimized by injection of each standard solution (1 μg/mL). The mass spectrometer was operated in the multiple reaction monitoring (MRM) mode for MS/MS measurements at the conditions detailed in Table 1. A waste valve diverted the column effluent to the mass spectrometer from 6 to 10 min in both modes. Data acquisition was performed with LabSolutions software 5.80 (Shimadzu, Kyoto, Japan).

### 4.7. Calibration and Quantitation

For the response curves constant amounts of internal standard (S) were mixed with varying amounts of analyte (A) in molar ratios between 0.1 and 10 (1:10, 1:5, 1:2, 1:1; 2:1, 5:1, 10:1). The concentrations ranged for D3G between 0.003 μg/L and 0.3 μg/L and for DON between 0.002 μg/L and 0.2 μg/L. All analytes applying SIDA had [^13^C]-labeled isotopologues. The response curves were calculated from molar ratios [n(S)/n(A)] versus peak area ratios [A(S)/A(A)] after LC-MS/MS measurement. Response functions were obtained using linear regression.

### 4.8. Sample Preparation

To 0.8 mL of degassed beer 4 mL acetonitrile and the internal standards [^13^C_6_]-D3G (30 μL of 1 μg/mL) and [^13^C_15_]-DON (20 μL of 1 μg/mL) were added. The samples were vortexed (20 s) and were completely applied on a Bond Elut Mycotoxin cartridge (500 mg, 3 mL, Agilent Technologies, Santa Clara, CA, USA). The liquids were passed through the cartridges by vacuum suction. The eluates were collected and evaporated until dryness. The samples were reconstituted with 200 μL acetonitrile/water (1/1, *v*/*v*) and membrane filtered (0.45 μm). Afterwards, LC-MS/MS analysis was performed.

### 4.9. Method Validation

#### 4.9.1. Limits of Detection and Quantitation

Beer free of the monitored mycotoxins was chosen as the blank matrix and used to determine LODs and LOQs as suggested by Vogelgesang and Hädrich [38]. Therefore, the blank matrix was spiked with the unlabeled analytes at four different amounts (3, 10, 20, and 30 μg/L for D3G and DON), each in triplicate.

#### 4.9.2. Recovery

Blank beer was spiked in triplicate with different amounts of analytes (10, 20, and 30 μg/L for D3G and DON) and analyzed as described previously. Recoveries were calculated as the ratio of detected and spiked contents.

#### 4.9.3. Precision

Naturally-contaminated beer with contents of D3G at 28 μg/L and DON at 15 μg/L was used for intra-day (*n* = 3) and inter-day (*n* = 3, within two weeks) precision measurements.

## Figures and Tables

**Table 1:** LC-MS/MS parameter of the stable isotope dilution assay of D3G and DON.

Analyte	Precursor Ion *m*/*z*	Product Ion *m*/*z*	Q1 Pre Bias (V)	Q3 Pre Bias (V)	CE (V)
D3G	457.3	427.2	17	13	16
		247.3	22	15	22
[^13^C_6_]-D3G	463.3	433.2	17	13	16
		247.3	22	15	22
DON	297.2	249.2	−21	−18	−12
		231.2	−20	−17	−12
[^13^C_15_]-DON	312.2	265.2	−21	−18	−12
		247.2	−20	−17	−12

**Table 2:** Validation data of the stable isotope dilution assay of D3G and DON.

Analyte	LOD (μg/L)	LOQ (μg/L)	Precision (*n* = 3) (RSD %)		Recoveries (%) at Spiking Levels of		
			Intraday	Interday	10 μg/L	20 μg/L	30 μg/L
D3G	2.99	8.84	0.5	5	112 ± 3	110 ± 3	105 ± 1
DON	1.49	4.44	0.3	7	97 ± 1	107 ± 2	108 ± 2

**Table 3:** Contents of D3G and DON in the analyzed beer samples.

Beer Type	Country	Alcohol Content (vol %)	D3G (μg/L)	DON (μg/L)	Molar Ratio n(D3G)/n(DON)
Lager	Germany	5.4	-	-	-
Wheat beer ^b^	Germany	5.1	10.6	20.3	0.34
Lager	Taiwan	5.0	15.3	6.43	1.54
Lager	China	4.7	8.31 ^a^	5.11	1.05
Lager	USA	5.0	20.1	14.1	0.92
IPA	USA	6.5	-	9.77	-
Triple Golden Ale	USA	8.0	18.1	9.40	1.25
Porter	USA	5.3	-	-	-
Belgian Style IPA	USA	8.3	63.3	28.3	1.45

- not detected; ^a^ below LOQ; ^b^ organic; IPA, Indian Pale Ale.

## Abbreviations

The following abbreviations are used in this manuscript:

D3G	Deoxynivalenol-3-glucoside
DON	Deoxynivalenol
ESI	Electrospray Ionization
HMBC	Heteronuclear Multiple Bond Correlation
LC-MS/MS	Liquid chromatography combined with tandem mass spectrometry
LOD	Limit of Detection
LOQ	Limit of Quantitation
NMR	Nuclear Magnetic Resonance
qNMR	quantitative NMR
SIDA	stable isotope dilution assay
SPE	solid phase extraction
